# First-line antibiotic therapy in *Helicobacter pylori*-negative low-grade gastric mucosa-associated lymphoid tissue lymphoma

**DOI:** 10.1038/s41598-017-14102-8

**Published:** 2017-10-30

**Authors:** Sung-Hsin Kuo, Kun-Huei Yeh, Ming-Shiang Wu, Chung-Wu Lin, Ming-Feng Wei, Jyh-Ming Liou, Hsiu-Po Wang, Li-Tzong Chen, Ann-Lii Cheng

**Affiliations:** 1 Department of Oncology, National Taiwan University, Taipei, Taiwan; 2 Department of Internal Medicine, National Taiwan University, Taipei, Taiwan; 3 Department of Pathology, National Taiwan University, Taipei, Taiwan; 4 Cancer Research Center, College of Medicine, National Taiwan University, Taipei, Taiwan; 5 Graduate Institute of Oncology, College of Medicine, National Taiwan University, Taipei, Taiwan; 6 National Institute of Cancer Research, National Health Research Institutes, Tainan, Taiwan; 7 Department of Internal Medicine, Kaohsiung Medical University Hospital, Kaohsiung Medical University, Kaohsiung, Taiwan; 8 Department of Internal Medicine, National Cheng-Kung University Hospital, Tainan, Taiwan; 9 National Taiwan University Cancer Center, College of Medicine, National Taiwan University, Taipei, Taiwan

## Abstract

First-line antibiotic treatment for eradicating *Helicobacter pylori* (HP) infection is effective in HP-positive low-grade gastric mucosa-associated lymphoid tissue lymphoma (MALToma), but its role in HP-negative cases is uncertain. In this exploratory retrospective study, we assessed the outcome and potential predictive biomarkers for 25 patients with HP-negative localized gastric MALToma who received first-line HP eradication (HPE) therapy. An HP-negative status was defined as negative results on histology, rapid urease test, ^13^C urea breath test, and serology. We observed an antibiotic response (complete remission [CR], number = 8; partial remission, number = 1) in 9 (36.0%) out of 25 patients. A t(11;18)(q21;q21) translocation was detected in 7 (43.8%) of 16 antibiotic-unresponsive cases, but in none of the 9 antibiotic-responsive cases (P = 0.027). Nuclear BCL10 expression was significantly higher in antibiotic-unresponsive tumors than in antibiotic-responsive tumors (14/16 [87.5%] vs. 1/9 [11.1%]; P = 0.001). Nuclear NF-κB expression was also significantly higher in antibiotic-unresponsive tumors than in antibiotic-responsive tumors (12/16 [75.0%] vs. 1/9 [11.1%]; P = 0.004). A substantial portion of patients with HP-negative gastric MALToma responded to first-line HPE. In addition to t(11;18)(q21;q21), BCL10 and NF-κB are useful immunohistochemical biomarkers to predict antibiotic-unresponsive status in this group of tumors.

## Introduction

Most low-grade gastric mucosa-associated lymphoid tissue lymphomas (MALT lymphomas) are characterized by close association with *Helicobacter pylori* (HP) infection, and eradication of HP can cure approximately 70% of these tumors^1–4^. In contrast to HP-positive gastric MALT lymphomas, the role of first-line antibiotics in the treatment of HP-negative gastric MALT lymphomas remains uncertain^4–8^. Previous sporadic reports have revealed that certain types of HP-negative gastric MALT lymphomas can respond to common regimens that are used for HP eradication (HPE) therapy, i.e., a proton-pump inhibitor (PPI) plus clarithromycin, amoxicillin, metronidazole, or other antibiotics^9–11^.

As mentioned in these reports, some HP-negative patients might still have HP-associated tumors because previous use of bismuth, PPIs, and antibiotics could lead to pseudo-negative results on conventional HP tests such as the rapid urease test, the urea breath test, and histology^12–14^. In addition, one cannot completely exclude the possibility of a false HP*-*negative status if histomorphological findings disclose atrophic gastritis or intestinal metaplasia^15,16^. Furthermore, infection with coccoid forms of HP, which are difficult to be cultured and detected by immunohistochemical staining, and produce less urease, may cause pseudo-negative results on HP tests^17–19^.

The t(1;14)(p22;q32) chromosomal translocation, juxtaposing the *BCL10* gene of chromosome 1p to the immunoglobulin gene locus of chromosome 14q, results in strong expression of a truncated BCL10 protein in the nuclei and cytoplasm in MALT lymphoma^1,20,21^. However, t(1;14)(p22;q32) is rarely found in gastric MALT lymphoma. Several studies have demonstrated that the t(11;18)(q21;q21) translocation can predict HP independence (tumor unresponsive to HPE) in patients with HP-positive gastric MALT lymphoma^21–24^. Furthermore, the presence of t(11;18)(q21;q21) translocation was more frequently found in HP-negative gastric MALT lymphoma than in HP-positive gastric MALT lymphoma^7,25,26^. Previously, we showed that regardless of the status of the t(11;18)(q21;q21) translocation, nuclear expression of BCL10 and NF-κB is closely associated with HP independence in gastric MALT lymphoma^3,27^.

Epidemiologic studies have shown that the presence of cytotoxin-associated gene A (CagA) protein, the most important HP virulence factor, is associated with the formation of lymphoid follicles and MALT lymphoma of the stomach^28,29^. Previous studies reported that the CagA-seropositive rate in patients with gastric MALT lymphoma ranged from 89% to 96%^30,31^.

Lehours *et al*. reported detection of the *CagA* gene in 47.4% of the HP strains obtained from 90 cases of gastric MALT lymphoma^32^. Among t(11;18)(q21;q21)-negative gastric MALT lymphoma cases, Sumida *et al*. found that titers of anti-CagA were significantly higher in HP-dependent cases than in HP-independent cases^33^. We recently found that 11 HP strains isolated from patients with HP-dependent gastric lymphomas (5 gastric diffuse large B-cell lymphomas with histologic evidence of MALT and 6 gastric MALT lymphomas) were *CagA* positive^34^. We and other investigators demonstrated that CagA can promote cellular proliferation and attenuate apoptosis of B-cells through activation of CagA-signaling such as SRC homology-2 domain-containing phosphatase (SHP2) and extracellular signal-regulated kinase (ERK)-related signaling, or BAD phosphorylation and p53 accumulation^35–38^. Furthermore, we reported that HP CagA protein and its signaling pathway proteins, such as phospho (p)-SHP2, p-ERK, p-38 mitogen-activated protein kinase (MAPK), BCL-2, and BCL-XL, can be detected in tumors of gastric MALT lymphoma^39,40^. The expression of CagA and CagA-signaling molecules is closely associated with HP-dependence of these tumors^40^, indicating CagA may serve as a marker for the presence of HP for gastric MALT lymphoma.

In this study, we assessed the response rate and the long-term disease-free status of patients with localized HP-negative gastric MALT lymphoma (all negative for histology [including HP, atrophic gastritis, and intestinal metaplasia], rapid urease test, ^13^C urea breath test, and serology as well as for CagA expression in tumor cells and gastric microenvironments) who received first-line HPE regimens consisting of PPIs plus clarithromycin and amoxicillin. We also investigated the association between potential biomarkers, including t(11;18)(q21;q21), nuclear BCL10 expression, and nuclear NF-κB expression, and antibiotic-unresponsive status of the same type of tumors.

## Results

### Clinicopathological features and tumor response to HP eradication therapy

**Figure 1:**
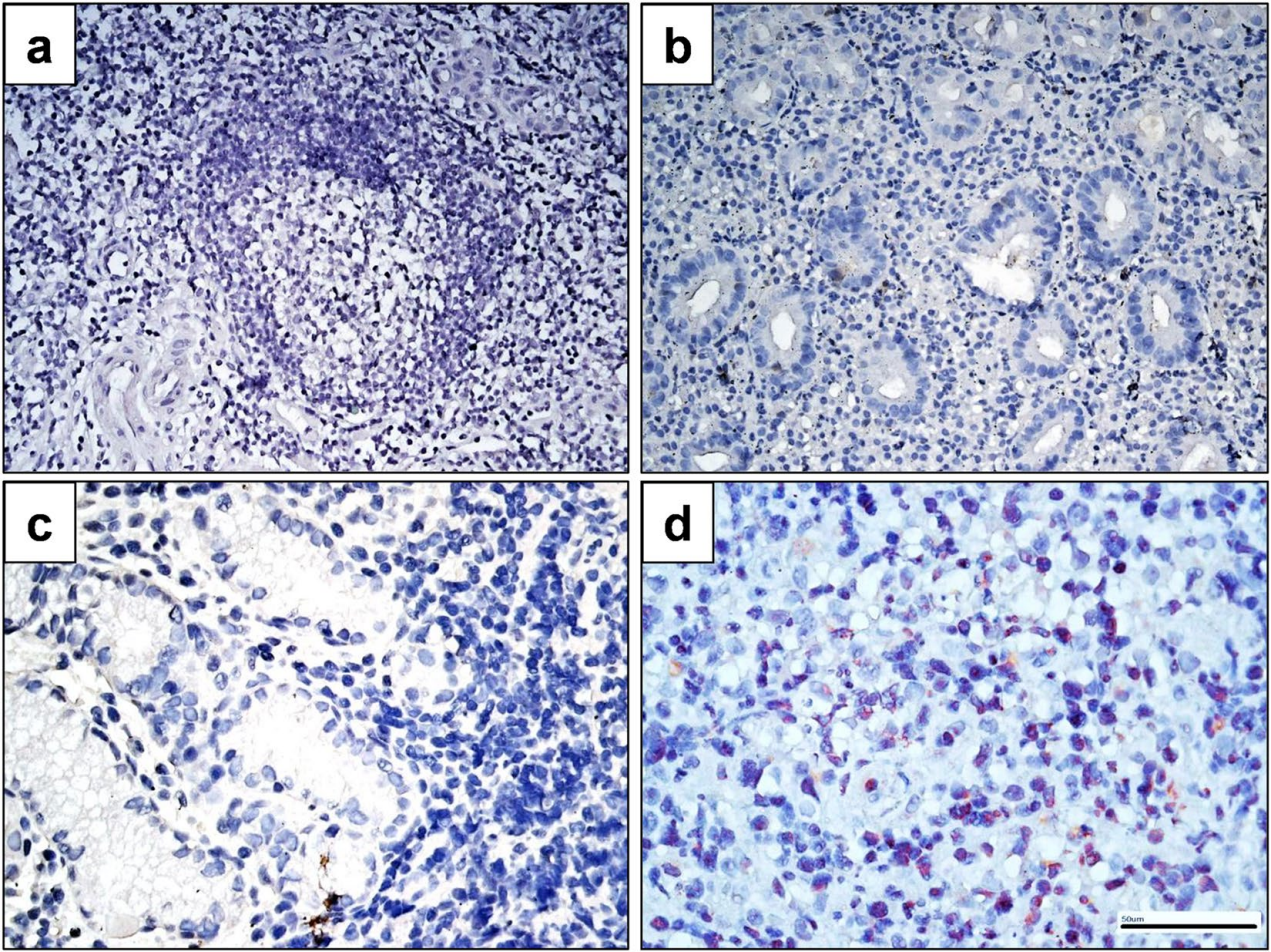
Immunohistochemical analysis of CagA expression in tumor cells of HP-negative gastric MALT lymphoma. (**a**) An antibiotic-responsive case (Case 9#, time to complete remission [CR] after completing HPE, 5 months) displaying no CagA expression in the tumor cells of the gastric mucosa (**b**) An antibiotic-responsive case (Case 19#, time to CR after completing HPE, 10 months) displaying no CagA expression in the tumor cells of the gastric mucosa (**c**) An antibiotic-responsive case (Case 23#, time to CR after completing HPE, 4 months) displaying no CagA expression in the tumor cells of the gastric mucosa (**d**) An antibiotic-responsive case of HP-positive gastric MALT lymphoma (time to CR after completing HPE, 1 month) displaying nuclear CagA expression in the tumor cells of the gastric mucosa (served as positive control).

Between January 1, 2005, and June 30, 2014, 25 patients with newly diagnosed stage IE/IIE1 primary HP-negative (results of the histology [including HP, atrophic gastritis, and intestinal metaplasia], rapid urease test, ^13^C urea breath test, and serology were all negative) gastric MALT lymphoma who received HPE as first-line treatment were included. Among them, 18 cases were also negative for HP cultures. Furthermore, CagA expression was not detected in tumor cells of all patients, indicating that HP is not present in these 25 cases (Fig. 1). We also showed that there was no *CagA* gene detected in gastric tumor biopsies obtained from patients with antibiotic-responsive tumors (Supplementary method, data not shown). Among these, 22 (88.0%) were at stage IE and three (12.0%) were at stage IIE1 (Table 1). Regarding the underlying diseases, four patients had hepatitis B virus infection, one patient had a hepatitis C virus infection, and one patient had an autoimmune disease (Sicca syndrome). Twenty-one (84.0%) of the 25 patients were treated with amoxicillin, clarithromycin, and omeprazole, whereas 4 (16.0%) patients received amoxicillin, clarithromycin, and lansoprazole.

**Table 1:** Characteristics, first-line antibiotics responses, and second-line treatment responses of HP-negative gastric MALT lymphoma patients.

Case	Sex/Age	Stage	Response	Time to CR	2^nd^-line Tx/time to 2^nd^-line Tx after diagnosis	Response to 2^nd^-line Tx	Relapse	Survival*	Serum IFE**
1	M/54	I	PD		R-COP (6)/12.5 mos	CR	Parotid gland	+106 mos	NA
2	M/53	I	SD		Chlorambucil*/7 mos	SD		+102 mos	NO
3	F/49	I	SD		Chlorambucil/14 mos	CR		+98 mos	NO
4	M/65	I	SD		Chlorambucil/12 mos	CR		+46 mos	IgM/lambda
5	M/48	I	SD		Chlorambucil + Prednisolone/15 mos	CR	Stomach	+94 mos	NO
6	F/33	I	PD		Riuximab/43 mos	SD		+89 mos	NO
7	M/39	I	SD		Radiotherapy/12 mos	CR		+88 mos	NA
8	M/57	I	SD		Radiotherapy/12 mos	CR		+82 mos	NA
9	F/69	I	CR	5 mos				+75 mos	NO
10	F/20	I	SD		Radiotherapy/5 mos	CR		+65 mos	NA
11	F/76	I	CR	12 mos				+64 mos	NO
12	M/76	IIE	SD		Chlorambucil/12 mos	CR		+59 mos	NO
13	M/88	IE	CR	24 mos				+56 mos	NA
14	M/58	I	SD		Observation			+51 mos	NO
15	F/59	I	PR		Observation			+48 mos	NO
16	F/49	I	SD		Chlorambucil/11 mos	CR		+42 mos	NO
17	M/61	I	CR	15 mos				+36 mos	NO
18	M/62	I	PD		R-COP/18 mos	CR		+31 mos	IgM/lambda
19	M/61	I	CR	10 mos				+24 mos	NO
20	M/74	IIE1	SD		Chlorambucil/6 mos	CR		+23 mos	IgM/kappa
21	M/71	I	SD		Chlorambucil/8 mos	CR		+22 mos	NO
22	F/51	I	CR	1 mos				+21 mos	NO
23	F/77	I	CR	4 mos				+20 mos	NO
24	M/48	IIE1	SD		Observation			+18 mos	NO
25	F/32	I	CR	7 mos				+18 mos	NO

Abbreviation: HP, *H*. *pylori*; CR, complete remission; Tx, treatment; IFE, immunofixation electrophoresis; M, men; F, women; PD, progressive disease; SD, stable disease; PR, partial remission; mos, months; R, rituximab; COP, cyclophosphamide, vincristine, and prednisolone; NO, no monoclonal gammopathy; NA, no analyses. Case information: Hepatitis B virus carrier, cases #3, #6, #16, and #19; Sicca syndrome, case # 14; Hepatitis C virus infection, case #22. *Survival, alive with follow-up time after treatment (months). **Immunofixation electrophoresis(IFE) showed IgM or IgG monoclonal gammopathy.

The histological scoring system proposed by the Groupe d’Etude des Lymphomes de l’Adult (GELA) is currently recommended to improve the consistency between the findings of different studies regarding first-line HPE for gastric MALT lymphoma^41^. The European Gastro-Intestinal Lymphoma Study (EGILS) consensus and the International Extranodal Lymphoma Study Group (IELSG) study therefore recommend the routine use of the GELA criteria in evaluating the response to treatment, including HPE, of gastric MALT lymphoma^42,43^. A complete remission (CR) is defined by the GELA grading system as the total disappearance of gross lymphoma and a negative histologic finding (CR or probable minimal residual disease [pMRD]), whereas partial remission (PR) is defined as normalization or reduction of macroscopic findings, histologic signs of lymphoma regression, and no signs of progression^42,43^. Previously, Fishbach *et al*. reported that 32 (32%) of 101 patients with pMRD or PR of tumors (according to the GELA criteria) after successful HPE achieved a histologic CR and 62% of patients had stable disease during the second-year of follow-up^44^. Another international randomized LY03 trial showed that the addition of an alkylating agent, chlorambucil, did not result in a better recurrence/progression-free survival or overall survival for patients with gastric MALT lymphoma who responded to HPE (including a CR or PR) compared with those who received “watch and wait”^45^.

**Figure 2:**
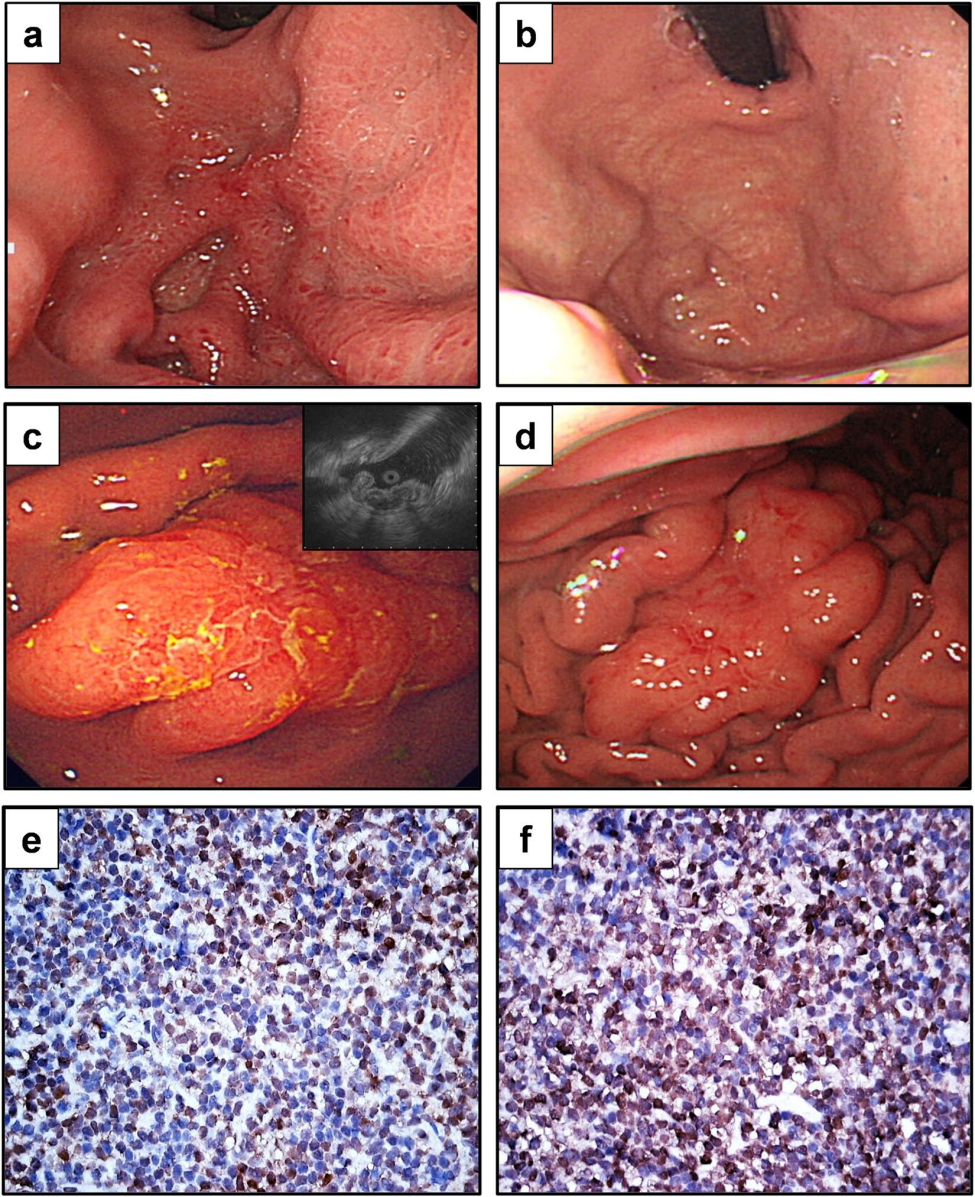
Endoscopic features and immunohistochemical analysis of BCL10 and NF-κB expression in HP-negative gastric MALT lymphoma. (**a**) Endoscopy showing a 2 cm slightly raised lesion with hyperemic patches in the antrum of the stomach of a 51-year-old woman (Case 22#). (**b**) One month after the completion of an HPE regimen, CR was achieved. (**c**) Endoscopy showing elevated and enlarged folds lesions measuring about 3~4 cm in the upper body of a 59-year-old woman. Right upper bottom, endoscopic ultrasound showing tumor with mucosa involvement (thickness up to 7.2 mm at second layer) (Case #15). (**d**) Four months after the completion of an HPE regimen, partial remission was achieved. (**e**) Nuclear expression of BCL10 in tumor cells of an antibiotic-unresponsive case (Case #6). (**f**) Nuclear expression of NF-κB in tumor cells of an antibiotic-unresponsive case (Case #6). HPE, H. pylori eradication therapy.

**Figure 3:**
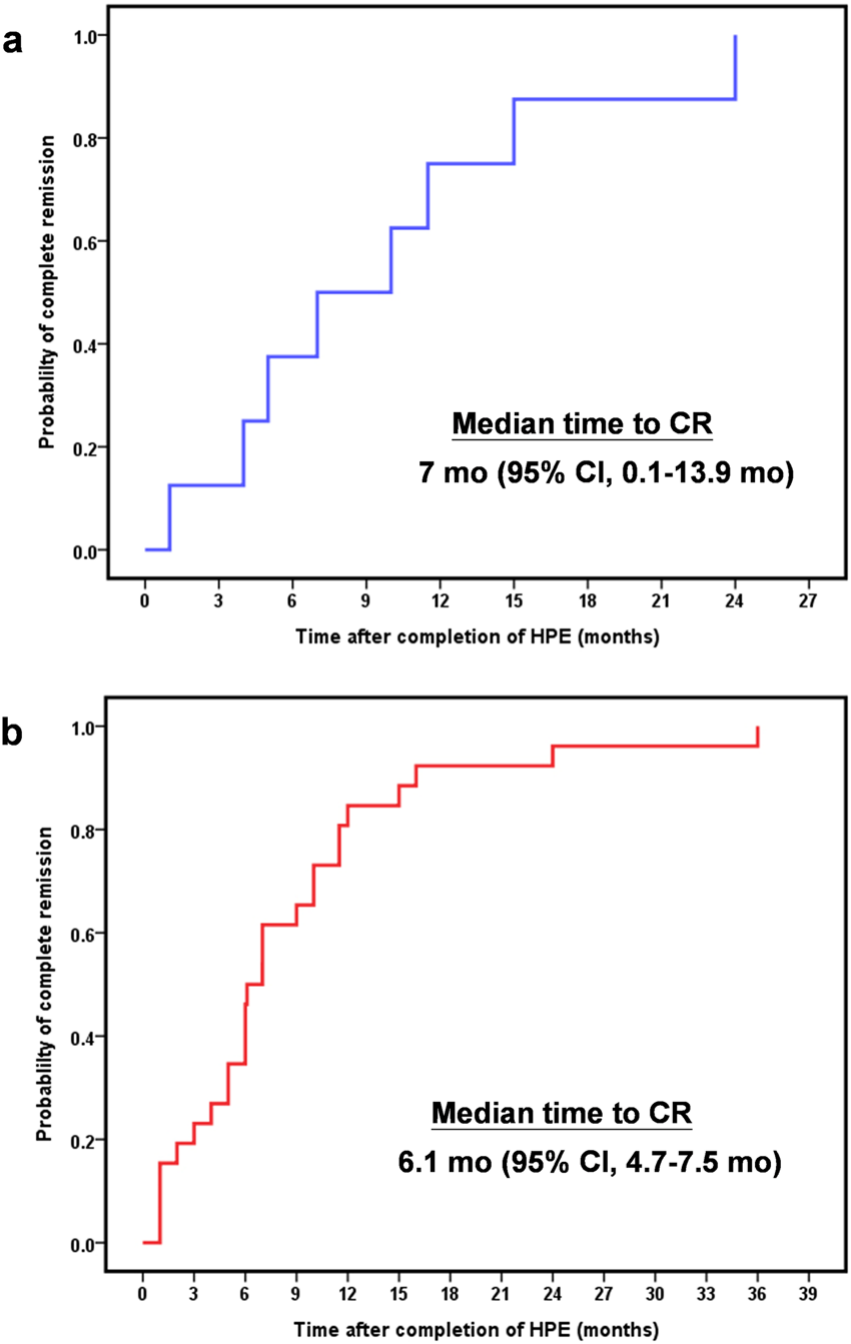
The time to complete remission of patients with antibiotic-responsive tumors. (**a**) Time to CR in our 8 cases was calculated from the completion of antibiotic treatment to the first evidence of CR through Kaplan–Meier analysis. (**b**) Time to CR in 26 cases of our series and that of five other investigators (refs^9,16,55,57,58^) was calculated from the completion of antibiotic treatment to the first evidence of CR through Kaplan–Meier analysis. Mo, month; CI, confidence interval; HPE, H. pylori eradication therapy.

Therefore, based on the GELA criteria for evaluating responses of gastric MALT lymphomas to HPE, we categorized our patients into two subgroups, those having antibiotic-responsive tumors (including CR and PR) and those having antibiotic-unresponsive tumors (including stable disease [SD] and progressive disease [PD]). The clinicopathological features of 9 patients (CR, number = 8; PR, number = 1) with antibiotic-responsive tumors (Fig. 2a–d) and 16 patients with antibiotic-unresponsive tumors, and the responses of their tumors to HPE are summarized in Table 1. We observed antibiotic responses in 9 (36.0%; 95% confidence interval [CI], 17.2–54.8%) out of 25 patients, and the median time to a CR (number = 8) was 7 months (95% CI, 0.1–13.9 months) (Fig. 3a). The antibiotic-responsive rate for 22 patients with stage IE was 40.9% (9/22), whereas the antibiotic-responsive rate for three patients with stage IIE1 was 0%. Of 20 patients receiving serum immunofixation electrophoresis (IFE) assessments, immunoglobulin M lambda monoclonal gammopathy was detected in 3 (25.0%) of the 12 antibiotic-unresponsive tumors, but not in the 8 antibiotic-responsive tumors (P = 0.242, Table 1).

Among the 16 cases without a tumor CR or PR, 14 patients who had persistent or increasing epigastric discomfort and were endoscopically or pathologically documented to have progressive tumors during the regular follow-up period were administered salvage treatments including oral alkylating agents (chlorambucil), rituximab-based regimens, and radiotherapy (Table 1). The time to aforementioned rescue therapy following HPE failure in these 14 patients is listed in Table 1. Of the 14 patients receiving second-line treatments, 12 patients achieved a CR while two patients had SD. Notably, two patients with SD undergoing observation alone experienced no progression during the follow-up (18 to 51 months after treatment) (Table 1).

At a median follow-up of 51.0 months (95% CI, 34.7–67.3 months), all patients with responsive tumors after HPE therapy were alive and free of lymphomas and progression, whereas 2 (16.7%) out of 12 patients with responsive tumors after second-line therapy experienced relapse (one in the parotid gland and the other in the stomach) (Table 1).

### Correlation of clinicopathological features, t(11;18)(q21;q21), and expression of BCL10 and NF-κB with tumor response to HP eradication therapy

In addition to the patient-related factors (sex, P = 0.087), the endoscopic appearance (ulceration or ulcerated mass, P = 0.671*)*, lesion sites (proximal location or 2-component involvement; P = 0.100), clinical stage (IIE1, P = 0.280), and endoscopic staging (tumors extending into the muscularis propria or beyond; P = 0.345) were not associated with the antibiotic-unresponsive status **(**Table 2 **)**.

**Table 2:** Correlation between clinicopathologic features, t(11;18)(q21;q21), and nuclear expression of BCL10 and NF-κB with tumor response to HP eradication therapy in HP-negative gastric MALT lymphomas.

Clinicopathologic characteristics	Tumors response to HPE regimen		P*
	Antibiotic-responsive	Antibiotic-unresponsive	
	(no. = 9)	(no. = 16)	
Age (median, range, years)	61 (32–88)	53.5 (20–76)	
Sex, male/female	6-Mar	4-Dec	0.087
Stage, no. (%)			0.28
IE	9 (100%)	13 (81.2%)	
IIE1	0 (0.0%)	3 (18.8%)	
Endoscopic features, no. (%)			0.671
Ulceration, ulcerated mass, or giant nodular folds	5 (55.6%)	11 (68.8%)	
Gastritis-like or erosions on infiltrative mucosa	4 (44.4%)	5 (31.2%)	
Location of tumor(s), no. (%)			0.1
Proximal^a^ or ≥2 components	5 (55.6%)	10 (62.5%)	
Distal^b^	4 (44.4%)	6 (37.5%)	
Depth of gastric wall involvement, no. (%)¶			0.345
Submucosa or above	6/7 (85.7%)	9/16 (56.2%)	
Muscularis propria or beyond	1/7 (14.3%)	7/16 (43.8%)	
API2-MALT1, no. (%)			0.027
Negative	9 (100%)	9 (56.2%)	
Positive	0 (0.0%)	7 (41.2%)	
BCL10 expression, no. (%)			0.001
Cytoplasmic or negative	8 (88.9%)	2 (12.5%)	
Nuclear	1 (11.1%)	14 (87.5%)	
NF-κB expression, no. (%)			0.004
Cytoplasmic or negative	8 (88.9%)	4 (25.0%)	
Nuclear	1 (11.1%)	12 (75.0%)	
2^nd^-line treatment regimen			
Chlorambucil		8 (50.0%)	
Rituximan-based		3 (18.7%)	
Radiotherapy		3 (18.7%)	
Observation		2 (12.6%)	

Abbreviation; HPE, *H*. *pylori* eradication therapy; HP, *H*. *pylori*; no., number. Proximal^a^: Middle body, upper body, fundus, or cardia. Distal^b^: Antrum, angle, or lower body. P*: comparison of discrete variables between HP-dependent and HP-independent. *P* values (two sided) were calculated using Fisher’s exact test. ^¶^Gastric wall involvement was evaluated by endoscopic ultrasonography or computed tomography in 23 patients.

The API2-MALT1 fusion transcript of t(11;18)(q21;q21) was detected in 7 (43.8%) of the 16 antibiotic-unresponsive tumors, but not in the 9 antibiotic-responsive tumors (P = 0.027, Table 2). Nuclear BCL10 expression was significantly higher in the antibiotic-unresponsive group than in the antibiotic-responsive group (14 out of 16 [87.5%] vs. 1 out of 9 [11.1%]; P = 0.001) **(**Fig. 2e, Table 2 **)**. Similarly, nuclear NF-κB expression was detected in 12 (75.0%) out of the 16 antibiotic-unresponsive tumors and in 1 (11.1%) out of the 9 antibiotic-responsive tumors (P = 0.004) **(**Fig. 2f, Table 2 **)**. Nuclear BCL10 expression was also more frequently observed in t(11;18)(q21;q21)-positive tumors than in t(11;18)(q21;q21)-negative tumors (6 out of 7 [85.7%] vs. 9 out of 18 [50.0%]; P = 0.179). Furthermore, nuclear NF-κB expression was more frequently observed in t(11;18)(q21;q21)-positive tumors than in t(11;18)(q21;q21)-negative tumors (6 out of 7 [85.7%] vs. 7 out of 18 [38.9%]; P = 0.073).

## Discussion

In this study, we demonstrated that nine (36.0%) out of 25 patients with HP-negative gastric MALT lymphoma were responsive to HPE, and remained lymphoma-free and progression-free at the longest follow-up. Our findings are consistent with a systematic review of published articles that demonstrated that a first-line HPE regimen resulted in a CR rate of 15.5% in 110 patients with HP-negative gastric MALT lymphoma^10^. As an addition to Zullo *et al*.^10^, who analyzed the CR rate after first-line antibiotic treatment, the diagnostic methods for HP, and the administration of HP regimens, we assessed the time to CR, the potential markers, including clinical stage, t(11;18)(q21;q21), and BCL10 expression, in 22 published results from 1999 through 2016 (summarized in Table 3)^5–7,9,15,16,25,26,46–58^. Overall, including our report, the CR rate after completing HP eradication treatments was observed in 68 (27.9%) out of 244 patients. The most commonly used HPE regimens consisted of PPIs plus at least two antibiotics such as amoxicillin, clarithromycin, or metronidazole for 7 to 14 days. Examinations for the presence of HP were mostly based on positive results from histology, rapid urease tests, ^13^C urea breath tests, and serology, and culture and stool antigen tests had been evaluated in five studies^7,16,25,47,53^, including our study.

**Table 3:** Published reports on the efficacies of first-line antibiotics treatment in HP-negative gastric MALT lymphomas.

Author	Year	No. of patients	Stage	FU after Tx (mo)	CR rate No. (%)	Time to CR (mo)	Makers	HP test	HPE-like regimen
Steinbach *et al*.^5^	1999	6	IE	5 or more	0 (0)	ND	NA	H, RUT, S	A + C + P, C + T, T + M 21D
Nakamura T *et al*.^25^	2000	4	ND	ND	1 (25)	ND	t(11;18): 2/2 (+), non-CR	H, UBT, S, C	ND
Ruskone-Fourmestraux *et al*.^6^	2001	10	IE (6) IIE1 (4)	2–21 (8)	0 (0)	ND	NA	H, S, C, PCR	A + C + P for 14 days
Ye *et al*.^7^	2003	5	IE	4–12 (4.5)	0 (0)	ND	t(11;18): 2/5 (+), non-CR; BCL10 (N): 3/5 (+), non-CR	H, RUT, S, C	ND
Raderer *et al*.^46^	2006	6	IE	12–19 (17)	5 (83)	ND	Included in ref.^38^	H, UBT, S, SAT	C + M + P (7D)
Nakamura S *et al*.^15^^,#^	2006	7	IE(6) IIE1 (1)	1–15 (4)	2 (29)	ND	t(11;18): 0/1 CR; 3/3(+),non-CR	H, RUT, UBT, S	A + C + P with and without M
Akamatsu *et al*.^16^^,#^	2006	9	IE-IIE1	6 or more	1 (11)	6	NA	H, S, C	A + C + P (7D)
Nozaki *et al*.^47^	2006	1	IE	5 years	1 (0)		t(11;18): negative	H, UBT, C	A + C + P (7D)
Terai *et al*.^48^	2008	4	IE-IIE1	ND	1 (25)	ND	t(11;18): 3/3(+), non-CR	H, RUT, UBT, S	A + C + M + P (7D)
Nakamura T *et al*.^49^	2008	17	IE (16) IIE1 (1)	1 case: 43	2	ND	t(11;18): 1/2(+), CR; 7/15(+), non-CR	H, RUT, S	A + C + P with and without M
Dong *et al*.^50^	2008	1	IE	ND	0 (0)		NA	H, UBT, S	A + C + P for 28D
Stathis *et al*.^51^	2009	14	IE (9) IIE1 (5)	ND	5 (35)	ND	I: 5/9 (+), CR; II: 0/5 (+), CR	H, UBT, S	A + C + P, C + M + P, or A + M + P
Sumida *et al*.^33^	2009	9	IE	ND	0 (0)	ND	t(11;18): 4/9(+), non-CR; BCL10(N): 5/9 (+), non-CR	H, UBT, S	A + C + P
Park *et al*.^9^	2010	6	IE	27-Jun	3 (50)	2-Jan	NA	H, RUT, UBT, S	A + C + P (7 or 14D)
Asano *et al*.^52^	2012	17	IE (15) IIE1 (2)	0.3–12.7 years	5 (29)	ND	Single lesion or antrum location t(11;18):1/1(+), CR	H, RUT, UBT, S	A + C + P (16) (7D) A + M + P (1) (7D)
							6/9 (+), non-CR		
Nakamura S *et al*.^53^	2012	44	ND	ND	6 (14)	ND	NA	H, RUT, UBT, S, C	A + C + P, C + M + P, or A + M + P
Choi *et al*.^26^	2013	5	IE (4) IIE1 (1)	ND	2 (40)	ND	t(11;18): 0/2(+), CR; 2/3 (+), non-CR	H, RUT, UBT, S	A + C + P (7D)
									(7D)
Ryu *et al*.^54^	2014	9	IE-IIE1	ND	5 (56)	ND	NA	H, RUT, UBT, S	A + C + P (7 or 14D)
Raderer *et al*. ^46,55^	2015	13	IE (8)IIE1 (5)	42–181*	5 (46)	Mar-36	IE: 5/8 (+), CR; IIE1: 0/5 (+), CR; t(11;18): 0/5(+), CR 1/1(+), PR; 2/7(+), non-CR	H, UBT, S	C + M + P or C + A + P
									(7 or 14D)
Li *et al*.^56^	2016	4		ND	2 (50)		NA	H, UBT	A + C + M + P (7 or 14D)
Kim *et al*.^57^	2016	6		ND	3 (50)	6.1 (median)	NA	H, RUT, UBT	A + C + P (7 or 14D)
Gong *et al*.^58^	2016	28	IE (24)		16 (57)	11.5 (median)	NA	H, RUT, UBT, S	A + C + P (7 or 14D)
			IIE (1)/IV (3)						
Present study	2016	25	IE (22)	18–106	8 (32)	24-Jan	IE: 8/22 (+), CR; IIE1: 0/3 (+), CR; t(11;18): 0/7(+), CR 6/13 (+), non-CR; BCL10 (N): 13/16(+), non-CR; NF-κB (N): 11/16(+), non-CR	H, RUT, UBT, S, C	A + C + P (14D)
[kuo *et al*.]			IIE1 (3)						
**Overall**		**244** ^**&**^			**68 (27.9)**	**Median 6 mo**	**t(11;18): 39/74**(+)**, non-CR; 2/19**(+)**, CR; P** = **0.001**		

Abbreviation: No, number; FU, follow-up; Tx, treatment; Mo, months; CR, complete remission; HP, Helicobacter pylori; HPE, HP eradication;; ND, non-described; NA, non-analysis; t(11;18), t(11;18)(q21;q21); (+), positive; BCL(N), nuclear BCL10 expression; NF-κB (N), nuclear NF-κB expression. D, days. HP examination test: H, histology; RUT, rapid urease test; UBT, urea breath test; S, serological test; C, culture; SAT, stool antigen test; PCR, protein chain reaction. HPE-like regimen: A, amoxicillin; C, clarithromycin; M, metronidazole; T, tetracycline; P, proton-pump inhibitor, including lansoprazole, pantoprazole or esomeprazole for 7 to 21 days (D). ^#^Refs^15,16^, included 1 case with transformed high-grade MALT lymphoma, renamed as diffuse large B-cell lymphoma with MALT (DLBCL[MALT]). *Follow-up after diagnosis. ^&^Raderer *et al*.^55^: 13 patients (6 patients had been previously reported in ref.^46^.

Although the aforementioned published series (some series comprising less cases and some series comprising more cases than our present cases) demonstrating a CR rate of 27.4% (60/219) (Table 3), our current data underline the reality that a proportion of patients without evidence of HP infection can be cured by first-line HPE. First, we showed that none of our patients had histologic evidence of atrophic gastritis or intestinal metaplasia (the aforementioned histomorphological findings are clues of a previous HP infection) in their specimens before HPE^15,16^, even if they had undergone previous eradication therapy or antibiotics treatment. Second, we showed that none of our patients exhibited CagA expression in tumor cells or in the gastric microenvironment. We also showed that there was no *CagA* gene detected in gastric tumor biopsies obtained from patients with antibiotic-responsive tumors. These findings indicated that the CagA-negativity of the tumors of our cases is actually just another suggestion that it is a real HP-negative gastric MALT lymphoma. Third, we demonstrated that the time to response for patients with antibiotic-responsive tumors was 7 months (range: 1 to 24 months), and importantly, after the median long-term follow up of 51 months, all patients with responsive tumors were free of lymphoma or progression. Combining our results with that of five other investigators (Table 3)^9,16,55,57,58^, the median time to a CR for patients with HP-negative gastric MALT lymphoma who received first-line HPE was 6.1 months (95% CI, 4.7–7.5 months) (Fig. 3b).

In the current study, we reported that 29 (31.5%) of 93 patients with gastric MALT lymphoma were HP-negative. However, in a systematic review of gastric MALT lymphoma (including diffuse large B-cell lymphoma), Zullo *et al*. demonstrated that 117 (11.2%) of 1146 cases were negative for HP infection^59^. The decreased level of HP infection in the general population and the widespread use of antibiotics in treating gastric-related diseases may alter the epidemiology of gastric MALT lymphoma and contribute to the higher HP-negative rate in our population. Our results are consistent with previous studies that have reported an increase in the prevalence of HP-negative infection in gastric MALT lymphoma cases over the past decade. For example, Choi *et al*.^26^ reported a 19.7% HP-negative rate in 66 cases of gastric MALT lymphoma, in which patients received first-line antibiotics during a study of the assessment of remission rate and the incidence of API2-MALT1. Luminari *et al*. reported that the prevalence of HP infection in gastric MALT lymphoma decreased from 61% (cases diagnosed between 1997 and 2001) to 17% (cases diagnosed between 2002 and 2007)^60^. In a retrospective analysis of 97 cases of gastric MALT lymphoma, Raderer *et al*. showed an increased prevalence of HP-negative infection in cases diagnosed after 2004 compared with those diagnosed before 2004 (31.2% versus 18%)^55^. Mendes *et al*. also found that the prevalence of HP-negative infection in gastric MALT lymphoma diagnosed between 2005 and 2013 was 67.6% (25/37)^61^.

In addition to PPIs, the most commonly used antibiotics in the first-line treatment of HP in gastric MALT lymphoma include amoxicillin, clarithromycin, and metronidazole. In the present study, all patients received PPI plus clarithromycin and amoxicillin. It should be noted that increasing antimicrobial resistance of HP, especially for clarithromycin, has been observed in patients with HP infections worldwide^62–64^. In Europe, the prevalence of clarithromycin resistance by HP ranges from 5.6% to 36.6%^65^. In a nationwide study of primary resistance to HP in Taiwan after implementation of a national policy to restrict antibiotic consumption since 2001, Liu *et al*. showed that the prevalence of primary resistance to clarithromycin was 11.2% (95% CI 9.6–13%)^66^. In a similar time period, in the Asia-Pacific region, the prevalence of primary resistance to clarithromycin in China (2000–2009)^67^, Japan (2000–2013)^68^, and Korea (2009–2012)^69^ was 23.8% (69/290), 31.1% (334/1073), and 23.7% (27/114), respectively. Nevertheless, the prevalence of antimicrobial resistance of HP to clarithromycin in gastric MALT lymphoma is rarely studied because the sensitivity of HP culture is obviously lower than the sensitivity to histologic detection of HP in gastric MALT lymphoma^32^. Recently, Bilgilier *et al*. found that the rate of clarithromycin resistance in 13 cases of gastric MALT lymphoma was 15% through analyses of the HP 23S rRNA gene containing genotypic clarithromycin resistance^70^.

Although the question of why a certain proportion of HP-negative gastric MALT lymphomas may respond to antibiotics remains unanswered, several crucial findings may support the following speculations: (1) HPE regimens may also eradicate other bacteria or *Helicobacter*-like bacteria, such as *H*. *heilmannii* that is associated with the development of gastric MALT lymphoma in humans^71–73^. For example, Morgner *et al*. found that five patients with documented *H*. *heilmannii* infection achieved a CR after 14 days of omeprazole and amoxicillin therapy^72^. However, we cannot exclude that HPE regimens may eradicate intestinal microbiota that may be associated with the development of HP-negative gastric MALT lymphoma^74^. (2) In addition to eradicating HP and HP-like bacteria, clarithromycin has direct anti-neoplastic or immunomodulatory effects^46,55,75^. In a B-cell lymphoma cell line derived from a BALB/c mice model, O’Hara *et al*. showed that clarithromycin inhibited cell viability and induced apoptosis though down-regulating BCL-2 expression^76^. Mizunoe *et al*. showed that macrolides, either clarithromycin or azithromycin, caused apoptosis of activated lymphocytes through attenuation of BCL-XL expression^77^. Another macrolide, erythromycin, was found to have an inhibitory effect on proliferation of T-cells, and a possible mechanism is the down-regulation of NF-κB expression^78^. In CD4+ T-cells, azithromycin effectively inhibited cell proliferation and cytokine secretion through down-regulation of the activity of mammalian target of rapamycin^79^. The aforementioned immunosuppressive effect on CD4+ T-cells was also observed at a higher concentration of clarithromycin (40 mg/L)^79^. Clinically, Ishimatsu *et al*. reported two cases of pulmonary MALT lymphoma successfully treated using clarithromycin 200 mg per day^80^. Kiesewetter *et al*. reported another case of ocular adnexal MALT lymphoma achieving a CR after treatment with first-line clarithromycin 500 mg twice per day for 4 weeks^81^. Raderer *et al*. speculated that high-dose clarithromycin (500 mg twice a day for 14 days) may yield a better CR rate of 38.5% in their cases of HP-negative gastric MALT lymphoma^55^. Our study also revealed a CR rate of 32% in patients receiving a clarithromycin (500 mg twice a day for 14 days)-based regimen. Furthermore, one recently published phase II trial reported that high-dose clarithromycin (2 g a day for 14 days for each course) resulted in a CR rate of 26.9% in patients with relapsed or refractory extranodal MALT lymphoma^82^.

Previous studies have demonstrated that the API2-MALT1 fusion protein resulting from a t(11;18)(q21;q21) translocation can activate NF-κB through an API2 moiety-mediated auto-oligomerization and thus contribute to the HP-independent growth of gastric MALT lymphoma^83–85^. However, whether t(11;18)(q21;q21) can predict antibiotic unresponsiveness in HP-negative gastric MALT lymphomas remains unclear. A systematic review of published results (included our present study) revealed that the frequency of t(11;18)(q21;q21) was significantly higher in antibiotic-unresponsive tumors than in antibiotic-responsive tumors (39 out of 74 [52.5%] vs. 2 out of 19 [10.5%], P = 0.001) (Table 3). These findings also indicate that for the other 50% of antibiotic-unresponsive tumors, other predictive markers should be pursued.

In this study, we showed that nuclear expression of BCL10 or NF-κB is closely associated with an antibiotic-unresponsive status and that both molecules are associated with the status of t(11;18)(q21;q21). These findings are consistent with those of Ye *et al*.^7^ and Sumida *et al*.^33^ who reported on the relationship between an antibiotic-unresponsive status and BCL10 nuclear expression in an HP-negative gastric MALT lymphoma.

As shown in Table 3, notably, two cases of HP-negative gastric MALT lymphomas with t(11;18)(q21;q21) remained antibiotic-responsive^49,52^. In a large series of HP-positive gastric MALT lymphomas, Liu *et al*. also demonstrated that 2 (4.5%) out of 44 patients with t(11;18)(q21;q21) remained HP dependent^24^. In our series of HP-positive gastric MALT lymphomas (data not shown), the pivotal role of BCL10 or NF-κB in HP-independent growth^27,50,86,87^ was demonstrated by the finding that two cases with t(11;18)(q21;q21) but lacking both nuclear expression of BCL10 and NF-κB responded well to HPE (Supplementary Fig. 1)^87,88^. Of these two cases with t(11;18)(q21;q21)-positive but no nuclear NF-κB expressing tumors, one case harbored a fusion transcript of t(11;18)(q21;q21) that contained 3 intact BIR domains in the amino terminal API2 region, and an intact caspase-like domain, but none of the immunoglobulin-like domains in the carboxyl terminal MALT1 region. Previous studies showed that the fusion product of t(11;18)(q21;q21) comprising an intact immunoglobulin-like domain had a greater ability to stimulate NF-κB signaling than the fusion product without an intact immunoglobulin-like domain^89,90^. Several studies have demonstrated that t(11;18)(q21;q21)-mediated NF-κB activation requires an interaction between API2-MALT1 and TRAF2 or TRAF6^83–85^. The lack of an immunoglobulin-like domain and the disruption of the interaction with TRAF2 or TRAF6 of the API2-MALT1 fusion protein may be linked to the absence of nuclear NF-κB expression in some t(11;18)(q21;q21)-positive tumors that remain antibiotic-responsive.

In summary, the results of this study indicate that a substantial proportion of patients with early-stage HP-negative gastric MALT lymphoma remain antibiotic-responsive and can be cured using a first-line HPE regimen. In addition to t(11;18)(q21;q21), nuclear expression of BCL10 or NF-κB can help us predict antibiotics’ unresponsiveness. Further investigations into microbiota associated with the lymphomagenesis of HP-negative gastric MALT lymphoma are warranted.

## Patients and Methods

### Ethics statement

All experimental protocols were approved by the Institutional Review Board (IRB) of the Research Ethical Committee of National Taiwan University Hospital (NTUH IRB number: 9361700774). All experiments were conducted in accordance with the approved guidelines and regulations. The patients’ medical data were anonymized prior to access and analysis. All patients provided written informed consent to participate in and to provide tissue material for biological studies.

### Patients, treatment, and tissue samples

We screened study subjects from the Cancer Registry, Medical Information Management Office, and the lymphoma database of the Department of Pathology of the National Taiwan University Hospital in Taipei, Taiwan between January 1, 2005 and June 30, 2014. We identified 93 patients with stage IE/IIE1 gastric MALT lymphoma from patients diagnosed with primary gastric lymphoma. We retrospectively reviewed the medical records and pathologic records of these patients to evaluate whether these gastric MALT lymphomas were HP-positive or HP-negative tumors. Evidence of HP infection was defined as positive results on biopsy, histology, a urease test, a ^13^C urea breath test, or serology^91–93^. An HP-negative status was defined as total negative results on histology (included HP, atrophic gastritis, and intestinal metaplasia)^15,16^, a rapid urease test, a ^13^C urea breath test, and serology.

There were 63 patients with HP-positive tumors and 29 patients with HP-negative tumors. Since gastric MALT lymphoma is relatively indolent and pseudo-negative HP tests may occur, most HP-negative patients in our institution were treated with first-line HPE regimens, particularly if their symptoms were insignificant.

From complete medical records, among 29 patients with HP-negative gastric MALT lymphoma, two patients received radiotherapy, 2 patients received alkylating agents-based chemotherapy, and 25 patients received antibiotics as a first-line treatment. The antibiotics regimens were the same as the HPE regimen, which consisted of 500 mg of amoxicillin administered four times a day (or 1000 mg of amoxicillin administered twice a day), 500 mg of clarithromycin administered twice a day, and 20 mg of omeprazole or 30 mg lansoprazole administered twice a day for 2 weeks as first-line treatment.

Diagnosis of gastric MALT lymphoma was made according to the histological criteria described by Isaacson *et al*. and the European Gastro-Intestinal Lymphoma Study consensus report on gastric extranodal marginal zone B-cell MALT lymphoma^42,94^. The tumors were staged and classified according to the Musshoff modification of the Ann Arbor staging system. The patients also received an examination for the presence of monoclonal gammopathy using serum IFE. The patients underwent their first follow-up after an upper gastrointestinal endoscopic examination or an ultrasonic endoscopic examination 4 to 8 weeks following HPE. This examination was repeated every 12 to 16 weeks until we observed histological evidence of remission.

The regression of the tumor following HPE was histologically evaluated according to the criteria of the (GELA) histological scoring system^42,43^. A CR is defined by the GELA grading system as the total disappearance of gross lymphoma and a negative histologic finding (CR or pMRD), whereas PR is defined as normalization or reduction of macroscopic findings, histologic signs of lymphoma regression, and no signs of progression. Tumors that resolved to a CR or PR after HPE were considered antibiotic-responsive^42,43^. Two subgroups of patients were considered antibiotic-unresponsive: (1) those who had SD but failed to show histologic regression 24 months following HPE, and (2) those with tumors exhibiting objective evidence of PD at any time during the follow-up^42,43^.

### Multiplex reverse transcription polymerase chain reaction for the API2-MALT1 fusion transcript of t(11;18)(q21;q21) in lymphoma cells

Total cellular RNA was extracted from formalin-fixed and paraffin-embedded tissues using an Ambion RNA isolation kit (RecoverAll™ Total Nucleic Acid Isolation Kit, Ambion® | Life Technologies) and was analyzed for the API2-MALT1 fusion transcripts of the t(11;18)(q21;q21) translocation using multiplex reverse transcription polymerase chain reaction, followed by sequencing as described previously^27,95,96^. Gastric MALT lymphoma samples with API2-MALT1 fusion transcripts served as positive controls.

### Immunohistochemistry

Immunohistochemistry for BCL10 (sc-9560; Santa Cruz Biotechnology, Santa Cruz, CA, USA), NF-κB (p65; sc-109; Santa Cruz Biotechnology), and CagA (A10; sc-28368, Santa Cruz Biotechnology) was performed on paraffin-embedded sections of pre-HPE endoscopic biopsies, using an indirect immunoperoxidase method according to the manufacturer’s instructions^27,39,96^. Paraffin sections with the first, second, or both primary antibodies omitted were used as negative controls to verify the specificity of the staining.

The percentages of positive cells were averaged to yield an immunohistological score of 0–100%. The staining was considered positive for BCL10 and NF-κB (p65) if the protein was detected in more than 10% of the tumor cells; nuclear staining was performed according to the criteria described by Ye *et al*.^97^ and Oshima *et al*.^98^. For the CagA maker, positive expression was defined as ≥10% of cells with moderate or strong immunostaining (tumor cells with readily appreciable brown staining distinctly marking the tumor cell nucleus or cytoplasm), as previously described^39,40^.

### Statistical analysis

In this study, the Fisher exact test was used to compare the clinical characteristics, the presence of t(11;18)(p21;q21) as well as the expression levels of BCL10 and NF-κB (p65) between the antibiotic-responsive and antibiotic-unresponsive cases. The analyses were conducted using follow-up data that became available on December 31, 2015. Differences between the results of the comparative tests were considered statistically significant if the two-sided P-value was <0.05.

## Electronic supplementary material

Supplementary Methods and Supplementary Figure 1
