# Supplementary material for: First-line antibiotic therapy in Helicobacter pylori-negative low-grade gastric mucosa-associated lymphoid tissue lymphoma
Source: Sci Rep. 2017 Oct 30;7:14333. doi: 10.1038/s41598-017-14102-8 (PMC5662601; doi:10.1038/s41598-017-14102-8)
Supplement: Supplementary file 1 — Supplementary Methods and Supplementary Figure 1 [file 41598_2017_14102_MOESM1_ESM.pdf]

First-line antibiotic therapy in *Helicobacter pylori*-negative low-grade gastric mucosa-associated lymphoid tissue lymphoma

Sung-Hsin Kuo, Kun-Huei Yeh, Ming-Shiang Wu, Chung-Wu Lin, Ming-Feng Wei, Jyh-Ming Liou, Hsiu-Po Wang, Li-Tzong Chen, and Ann-Lii Cheng

Supplementary Methods

Interphase fluorescence in situ hybridization for t(11;18)(q21;q21)

The presence of t(11;18)(q21;q21)/API2-MALT1 on 4 mm formalin-fixed, paraffin-embedded (FFPE) sections was determined using the commercial probe (Vysis LSI/Abbott, *BIRC3/MALT1* dual-color, dual-fusion translocation prob. The method of interphase FISH was according to the manufacturer instructions.[86,87]

Detection of *cagA gene* by polymerase chain reaction analysis

Genomic DNA was extracted from pre-HPE FFPE specimens using an EX-WAX FFPE DNA extraction kit (S4530; *Chemicon*, Temecula, CA). The 3’ variable region of CagA gene was amplified using a 5'-CCTAGTCGGTAATGGGTTAT-3' forward primer *cagF* and a 5'-TTAATGCGTGTGTGGCTG -3' reverse primer *cagR*.[34]

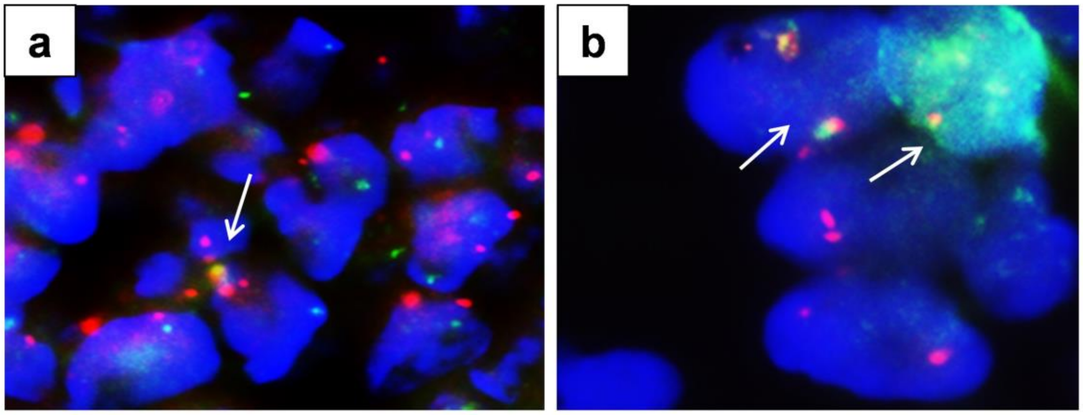

Supplementary Figure 1. Interphase fluorescence in situ hybridization for API2-MALT1 in two HP-dependent gastric MALT lymphoma cases. (**a**) Interphase fluorescence in situ hybridization with a *BIRC3/MALT1* dual-color, dual-fusion translocation probe showed colocalization of the red (*MALT1*) and green (*BIRC3*) signals (white arrow) in a HP-dependent case without nuclear expression of BCL10 and NF-κB. (**b**) Interphase FISH showed 2 fusion signal patterns (white arrows) of t(11;18)(q21;q21)/API2-MALT1 in another HP-dependent case without nuclear expression of BCL10 and NF-κB.
